# Outstanding Reviewers for *Nanoscale Advances* in 2025

**DOI:** 10.1039/d6na90039a

**Published:** 2026-06-19

## Abstract

We would like to take this opportunity to thank all of *Nanoscale Advances*’ reviewers for helping to preserve quality and integrity in chemical science literature. We would also like to highlight the Outstanding Reviewers for *Nanoscale Advances* in 2025.

## Abstract 2

We would like to take this opportunity to highlight the Outstanding Reviewers for *Nanoscale Advances* in 2025, as selected by the editorial team for their significant contribution to the journal.

## Graphical abstract

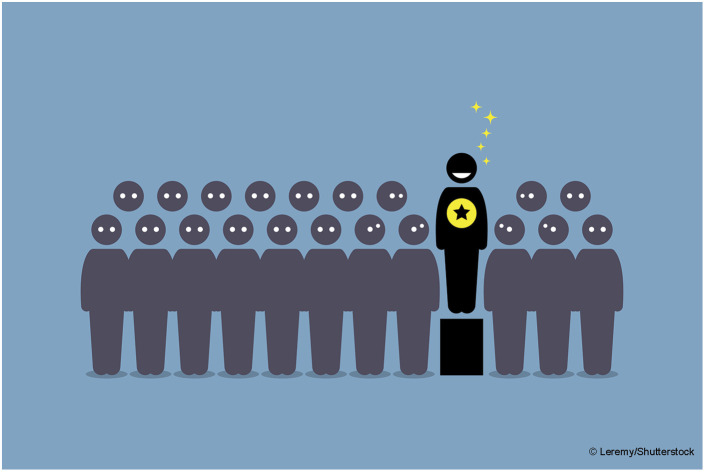

Each one of our outstanding peer reviewers has been carefully selected by our editorial team and includes active researchers who have made significant contributions to peer review and have gone above and beyond in their actions.

“*We extend our thanks to all of Nanoscale Advances’ reviewers for their invaluable contributions and are especially delighted to recognize this year’s Outstanding Reviewers for their exceptional dedication and support of the journal.*” – Professor Dirk M. Guldi, Editor-in-Chief

*“It is our great privilege to honor the outstanding reviewers. Their invaluable participation constitutes the cornerstone of the journal’s sustained development and ensures its high-standard academic quality. We hereby extend our sincere respect and gratitude to all of our reviewers for their unwavering professionalism and dedicated commitment to peer review endeavors.”* – Professor Yue Zhang, Editor-in-Chief

In recognition of the varied contributions of our reviewer community, our Outstanding Reviewers from 2025 have been chosen based on several different measures, including the number, timeliness and quality of the reports completed over the last 12 months. In addition to reviewers who provided a high number of quality reports, we are pleased to highlight reviewers who provided exceptionally thorough and detailed reports, reviewers who provided clear and insightful adjudicative reports and reviewers who made additional efforts to aid authors in improving their manuscripts.

Dr Carlos de Oliveira Amorim

University of Aveiro

ORCID: 0000-0003-4148-4374

Professor Xijun Liu

Guangxi University

Dr Zhongyu Liu

Stanford University

ORCID: 0000-0002-2777-8360

Dr Md Rizwanullah

Chitkara University

ORCID: 0000-0003-1394-7298

Dr Giovanni Marco Saladino

Stanford University

ORCID: 0000-0002-6854-1423

Dr Zhen Xiao

Stanford University

ORCID: 0000-0002-3740-3546

Dr Haoqing Xu

Duke University

ORCID: 0009-0007-9608-2272

Dr Hui Xu

Nanjing Tech University

ORCID: 0000-0002-9232-9625

We would also like to thank the *Nanoscale Advances* Editorial Board and Advisory Board and the nanoscience community for their continued support of the journal, as authors, reviewers and readers.

We continue to work on improving the diversity of our reviewer pool to reflect the diversity of the communities that we serve.

Professor Dirk M. Guldi, Editor-in-Chief

Professor Yue Zhang, Editor-in-Chief

Dr Jeremy Allen, Executive Editor

